# CSF markers of vascular injury correlate with tau and cognitive decline in early Alzheimer's disease

**DOI:** 10.1002/alz.70957

**Published:** 2025-11-30

**Authors:** J. Scott Miners, Gargi Roy, Seth Love, and for the Alzheimer's Disease Neuroimaging Initiative

**Affiliations:** ^1^ Cerebrovascular and Dementia Research Group, Translational Health Sciences, Bristol Medical School University of Bristol Bristol UK

**Keywords:** Alzheimer's disease, angiopoietin‐2, angiotensin‐converting enzyme‐1 (ACE‐1), blood–brain barrier, cerebrospinal fluid, endothelial, mild cognitive impairment, pericyte, placental growth factor, soluble platelet‐derived growth factor receptor beta (sPDGFRβ)

## Abstract

**INTRODUCTION:**

Cerebrovascular injury is common in Alzheimer's disease (AD), but its timing in relation to Aβ and tau pathology and cognitive decline remains unclear.

**METHODS:**

We measured baseline vascular marker levels in cerebrospinal fluid (CSF) and serum from 75 Alzheimer's Disease Neuroimaging Initiative (ADNI) study participants, stratified into cognitively unimpaired (CU), mild cognitive impairment (MCI), and AD groups (*n* = 25/group) and investigated associations with disease pathology (CSF and positron emission tomography [PET] amyloid beta [Aβ] and tau) and cognition (Clinical Dementia Rating scale [CDR], Montreal Cognitive Assessment, Mini‐Mental State Examination, and Alzheimer's Disease Assessment Scale).

**RESULTS:**

CSF markers of endothelial (placental growth factor, angiopoietin 2, angiotensin‐converting enzyme‐1 [ACE‐1]) and pericyte (soluble platelet‐derived growth factor receptor beta [sPDGFRβ]) injury were elevated in AD. Most were also higher in CDR 0.5 than CDR 0 and correlated with CSF tau and cognitive impairment in CU and MCI groups, particularly in PET Aβ‐positive (Aβ+) participants. Serum sPDGFRβ, tyrosine kinase with immunoglobulin and epidermal growth factor homology domains‐2 (TIE‐2), and ACE‐1 correlated with CSF measurements.

**DISCUSSION:**

Cerebrovascular injury precedes the development of dementia and, particularly in PET Aβ+ individuals, progresses in close association with CSF tau and cognitive decline.

**Highlights:**

- We measured the levels of multiple markers of neurovascular injury in serum and CSF taken at baseline from CU, MCI, and AD participants in the ADNI study and investigated associations with CSF and PET markers of disease pathology and with cognitive decline.
- CSF markers of neurovascular injury, particularly PlGF, are elevated in very early stages of AD, including in MCI and in PET Aβ+ CU individuals. The levels are closely related to CSF t‐tau and p‐tau and to cognitive decline
- Levels of only a few neurovascular markers in serum correlate with those in CSF: sPDGFRβ, TIE‐2, and ACE‐1.

## 1 BACKGROUND

Alzheimer's disease (AD) is characterized by amyloid beta (Aβ) plaque and neurofibrillary tangle pathology, but cerebrovascular pathology, including sporadic cerebral amyloid angiopathy (reviewed in Greenberg et al.^1^) and cerebral small vessel disease (reviewed in Kim et al.^2^), is very often also present. Disease modeling has suggested that vascular dysfunction precedes Aβ deposition and tau pathology^3^ and may occur up to 20 years before the onset of clinical symptoms. Reduced cerebral blood flow (reviewed in Korte et al.^4^) and blood–brain barrier (BBB) leakiness (reviewed in Barisano et al.^5^) precede and predict cognitive decline in the early stages of AD. Neuropathological studies have provided evidence that AD neuropathological change (ADNC) is accompanied by biochemical changes reflecting a widespread reduction in cerebral oxygenation that is associated with elevated levels of endothelin‐1 (a potent vasoconstrictor), pericyte degeneration, and BBB leakiness.^6^, ^7^, ^8^, ^9^ Recent single‐nucleus RNA studies on microvessel‐enriched samples of human *post mortem* brain tissue revealed that genome‐wide associated study (GWAS)‐associated AD risk genes are enriched within endothelial cells, in addition to microglia, and that endothelial and pericyte gene expression is dysregulated in AD.^10^, ^11^ Together, the studies provide evidence of a bidirectional relationship between cerebrovascular dysfunction and disease pathogenesis as a core pathological feature in AD.

The temporal relationship between cerebrovascular dysfunction, ADNC, and cognitive decline remains unclear. BBB leakiness in the hippocampus was reported in early‐stage AD, that is, Clinical Dementia Rating scale (CDR) = 0.5, independently of Aβ and tau pathology.^12^, ^13^ Cerebrospinal fluid (CSF) levels of endothelial injury markers, such as intercellular adhesion molecule 1 (ICAM‐1), vascular cell adhesion molecule 1 (VCAM‐1), and placental growth factor (PlGF), correlated strongly with cortical thinning and cognitive decline, and these relationships were more pronounced in positron emission tomography (PET) Aβ‐positive (Aβ+) participants.^14^ CSF angiotensin‐converting enzyme‐1 (ACE‐1), predominantly originating from endothelial cells within the brain, was elevated in AD.^15^ In lumbar CSF, the level of soluble platelet‐derived growth factor receptor beta (sPDGFRβ), released from injured pericytes, was higher in AD and correlated strongly with CSF tau level.^16^ CSF sPDGFRβ increased before the development of dementia, particularly in people with progressive mild cognitive impairment (MCI), and correlated with subsequent changes in Mini‐Mental State Examination (MMSE) scores.^17^, ^18^ CSF sPDGFRβ also correlated with markers of BBB leakiness, as assessed by Qalb,^17^, ^19^ and contrast‐enhanced magnetic resonance imaging (MRI),^12^ and with markers of neuroinflammation and neuronal injury.^20^ CSF levels of angiopoietin‐2 (ANGPT2), a decoy agonist within the ANGPT/tyrosine kinase with immunoglobulin and epidermal growth factor homology domains (TIE) signaling pathway, associated with vascular instability, was found to be elevated in MCI, correlating strongly with CSF phosphorylated tau (p‐tau) levels and with markers of BBB leakiness, neuroinflammation, and neuronal injury.^21^

CSF sPDGFRβ levels were elevated in MCI^17^ and AD^16^ and correlated with CSF total tau (t‐tau) and p‐tau levels ^16^, ^17^, ^18^, ^22^ and Qalb^18^, ^19^ across the AD spectrum. Likewise, CSF markers of endothelial injury, including ICAM‐1 and VCAM‐1, were elevated in preclinical AD in relation to CSF tau, cortical thinning, and cognitive decline.^14^ A recent study revealed that CSF markers of angiogenesis were specifically related to CSF p‐tau181 levels, cortical thickness, and cognitive decline.^23^ We previously showed that sPDGFRβ levels in serum from healthy donors correlated positively with those in matched CSF samples from the same donors^16^; serum and CSF ANGPT2 levels also correlated positively, and serum ANGPT2 correlated with Qalb.^24^ The pro‐angiogenic cytokine PlGF is elevated in both MCI and AD.^25^ PlGF signaling via vascular endothelial growth factor receptor 1 (VEGFR1) mediates a variety of cellular processes and can displace vascular endothelial growth factorA (VEGF‐A) from VEGFR1 to enhance classical VEGFR2‐mediated angiogenesis.^26^, ^27^

In this study, we examined how markers of endothelial and pericyte injury and BBB leakiness, in paired CSF and serum samples from a subset of participants in the Alzheimer's Disease Neuroimaging Initiative (ADNI) study who were CU or had mild cognitive impairment (MCI) or AD, varied in relation to markers of AD‐related neuropathological change (ADNC) and cognitive function. We explored the hypothesis that cerebrovascular injury would be evident in early‐stage AD and be closely related to ADNC and cognitive decline. We also investigated whether altered levels of markers of vascular injury in serum mirrored changes in matched CSF samples. Our study provides further evidence that altered CSF levels of neurovascular markers are closely related to BBB leakiness, tau changes, and cognitive decline in individuals who are Aβ+. Serum levels of most vascular injury markers did not generally reflect those in CSF; however, serum sPDGFRβ, TIE‐2, and ANGPT2 correlated weakly with markers of ADNC, BBB leakiness, and memory.

## 2 METHODS

### 2.1 Study cohort

CSF and serum were obtained at baseline from 75 participants in the ADNI study who were stratified into cognitively unimpaired (CU) controls, MCI, and AD (*n* = 25/group). Baseline CSF: Aβ_1‐42_, Aβ_1‐40_, t‐tau, and p‐tau levels and imaging biomarkers: PET‐Aβ (Centiloids) and PET‐tau (entorhinal standardized uptake value ratio [SUVR]) were obtained from the ADNI study. AD cases were selected based on established cut‐off values for CSF markers of AD pathology: t‐tau > 400 pg/mL, p‐tau > 60 pg/mL, and Aβ_1‐42 _< 550 pg/mL, according to Hansson et al.^28^ MCI cases were selected based on a clinical diagnosis of MCI‐AD and intermediate CSF Aβ and tau levels. CU controls did not have a clinical diagnosis of dementia and had CSF Aβ and tau within normal ranges. The age at baseline, gender, *APOE* ε4 carrier status, and mean CSF Aβ_1‐40_, Aβ_1‐42_, t‐tau, and p‐tau concentrations, and PET Aβ and tau levels in the three diagnostic groups are summarized in Table 1. All participants underwent detailed cognitive assessment, including Clinical Dementia Rating for memory (CDR‐M), global (CDR‐G), and sum of boxes (CDR‐SB); Montreal Cognitive Assessment (MoCA); Mini‐Mental State Examination (MMSE); Alzheimer's Disease Assessment Scale–Cognitive Subscale (ADAS‐COG), and extended ADAS‐COG‐13. Cognitive assessment scores at baseline were obtained from the ADNI study.

**TABLE 1 alz70957-tbl-0001:** Summary of demographic, pathological, and cognitive features of study cohort.

Demographics	CU	MCI	AD	*p*
Number	25	25	25	–
Age	69.5 ± 5.3	71.7 ± 6.9	73.0 ± 9.1	0.23
Male:Female	10:15	13:12	15:10	NS
APOE ε4 carriers, *n* (%)	2/25 (8)	14 (56)	18 (72)	*p* < 0.0001
**Pathology markers**	CU	MCI	AD	
CSF Aβ1‐40 (pg/mL)	17,549.6 ± 4493.0	18,240.0 ± 5179.9	18,880.8 ± 5336.7	NS
CSF Aβ42 (pg/mL)	1514.6 ± 574.2	964.8 ± 488.2	580.5 ± 179.4	*p* < 0.0001
CSF total tau (pg/mL)	183.1 ± 37.4	274.1 ± 73.6	405.6 ± 117.2	*p* < 0.0001
CSF p‐tau (pg/mL)	15.3 ± 2.9	26.4 ± 9.9	43.7 ± 16.5	*p* < 0.0001
PET Aβ+	0/24	18/25	25/25	*p* < 0.0001
PET Aβ (Centiloids)	1.9 ± 8.9	48.2 ± 37.6	107.5 ± 30.4	*p* < 0.0001
PET‐tau (SUVR)	1.1 ± 0.2	1.29 ± 0.1	1.6 ± 0.1	*p* < 0.0001
**Cognitive assessment**	CU	MCI	AD	
CDR‐M (0: 0.5: 1‐2)	24:01:00	10:14:01	02:11:12	*p* < 0.0001
CDR‐G (0: 0.5: 1‐2)	24:01:00	10:14:01	02:17:06	*p* < 0.0001
CDR‐SBR (mean ± SD)	0.0 ± 0.2	1.0 ± 1.4	2.8 ± 2.0	*p* < 0.0001
MoCA (mean ± SD)	26.3 ± 2.5	23.8 ± 4.6	19.0 ± 4.8	*p* < 0.0001
MMSE (mean ± SD)	29.3 ± 1.0	28.6 ± 1.7	23.8 ± 3.7	*p* < 0.0001
ADAS‐COG (mean ± SD)	4.9 ± 1.8	8.2 ± 5.8	16.8 ± 9.5	*p* < 0.0001
ADAS‐13 (mean ± SD)	7.1 ± 2.8	13.2 ± 8.7	25.7 ± 12.7	*p* < 0.0001

Abbreviations: Aβ, amyloid beta; AD, Alzheimer's disease; ADAS, Alzheimer's Disease Assessment Scale–Cognitive Subscale; APOE, apolipoprotein E; CDR‐M, Clinical Dementia Rating memory; CDR‐G, CDR global; CSF, cerebrospinal fluid; CU, cognitively unimpaired; MCI, mild cognitive impairment; MMSE, Mini‐Mental State Examination; MoCA, Montreal Cognitive Assessment; PET, positron emission tomography; SD, standard deviation; SUVR, standardized uptake value ratio.

Data used in the preparation of this article were obtained from the ADNI database (adni.loni.usc.edu). The ADNI was launched in 2003 as a public–private partnership, led by Principal Investigator Michael W. Weiner, MD. The primary goal of ADNI has been to test whether serial MRI, PET, other biological markers, and clinical and neuropsychological assessment can be combined to measure the progression of MCI and early AD.

**RESEARCH IN CONTEXT**

1. **Systematic review**: Recent studies suggest that CSF and possibly serum markers of endothelial and pericyte injury are related to BBB leakiness and tau pathology early in AD. We analyzed the relationships of CSF and serum neurovascular markers to cognition, Aβ, and tau in CU, MCI, and AD participants in the ADNI study.
2. **Interpretation**: CSF markers of endothelial (PlGF, ANGPT2, ACE‐1) and pericyte (sPDGFRβ) injury were elevated before the development of dementia, in MCI and in PET Aβ+ CU participants, and correlated with CSF tau and with cognitive decline. Serum levels of only a few of the markers correlated with measurements in CSF, but some correlated weakly with BBB leakiness, disease pathology, and cognitive decline.
3. **Future directions**: Longitudinal studies are needed to clarify the timing of cerebrovascular damage in relation to changes in cerebral Aβ and tau, particularly in at‐risk CU individuals.

### 2.2 sPDGFRβ ELISA

sPDGFRβ concentrations in CSF and serum were measured by sandwich ELISA (Invitrogen Catalog No.: EHPDGFRB, Thermo Fisher Scientific, UK). CSF samples (100 µL undiluted) and serum (diluted 1 in 10 in proprietary dilution buffer supplied with the kit) were measured in duplicate. Absorbance was read at 450 nM in a FLUOstar VANTI plate reader (BMG Labtech, Aylesbury, UK). Reproducibility reported in the datasheet indicates an interassay coefficient of variability (CV) < 12% and intra‐assay CV < 10%, with spike recovery between 90% and 110% for serum, plasma, and cell culture medium. sPDGFRβ concentrations in samples were calculated by interpolation against a standard curve derived from serial dilutions of recombinant sPDGFRβ (18,000–24 pg/mL).[Table alz70957-tbl-0001]

### 2.3 Angiopoietin‐2 (ANGPT2) ELISA

ANGPT‐2 concentration in CSF and serum was measured by ELISA (Quantikine kit, R&D Systems, UK). CSF was diluted two‐fold and serum five‐fold in a proprietary dilution buffer supplied with the kit. Absorbance was read at 450 nm in a FLUOstar VANTI plate reader (BMG Labtech). Measurements were made in duplicate, and concentrations of ANGPT‐2 were determined by interpolation against a standard curve generated by a serial dilution of recombinant ANGPT‐2 (3000–23.5 pg/mL).

### 2.4 TIE‐2 ELISA

TIE‐2 concentration was measured using a DuoSet Human TIE‐2 ELISA (R&D Systems, UK) according to the manufacturer's guidelines. Serum samples were diluted 1 in 50 in 1% PBS: BSA. CSF samples were used neat. Standards, samples, and blanks (1% PBS: BSA) were added in duplicate. Absorbance was read at 450 nM in a FLUOstar VANTI plate reader (BMG Labtech). TIE‐2 concentrations were interpolated from a standard curve, obtained from a two‐fold derails dilution of recombinat TIE‐2 (1000‐156.25 pg/ml).

### 2.5 Albumin ELISA

Albumin concentrations in CSF and serum were measured by sandwich ELISA (Catalog No.: 108788) (Abcam, Cambridge, UK). CSF samples were diluted 1 in 2000 and serum samples 1 in 500,000 in the proprietary dilution buffer supplied with the kit. Standards, samples, and blanks were added in duplicate. Absorbance was read at 450 nM in a FLUOstar VANTI plate reader (BMG Labtech). Albumin concentration was interpolated from a standard curve derived from serial dilution of recombinant human albumin (200–3.125 ng/mL).

### 2.6 PlGF Enzyme‐Linked Immunosorbent Assay (ELISA)

PlGF concentration in CSF and serum was measured by a high‐sensitivity ELISA (Quantikine kit, R&D Systems, UK) according to the manufacturer's guidelines. CSF samples were diluted 1 in 4, and serum 1 in 2, in the proprietary dilution buffer supplied with the kit. Standards, samples, and blanks were added in duplicate. Absorbance was read at 450 nM in a FLUOstar VANTI plate reader (BMG Labtech). The concentration of PlGF was interpolated against a standard curve, obtained by serial dilution of recombinant PLGF (200‐3.125 pg/mL). The mean values from duplicate measurements are provided.

### 2.7 VEGF‐A ELISA

VEGF‐A concentration was measured in serum using a DuoSet VEGF‐A ELISA kit (R&D Systems, UK) according to the manufacturer's guidelines. Serum samples were diluted 1 in 4 in 1% PBS: BSA. Standards, samples, and blanks were added in duplicate. Absorbance was read at 450 nM in a FLUOstar VANTI plate reader (BMG Labtech). The concentration of VEGF‐A was interpolated against a standard curve, obtained by serial dilution of recombinant VEGF‐A supplied with the kit (2000–31.25 pg/mL). The mean values from duplicate measurements are provided.

### 2.8 VCAM‐1 ELISA

VCAM‐1 concentration was measured in CSF and serum by using the DuoSet VCAM1 ELISA kit (R&D Systems, UK) according to the manufacturer's guidelines. Serum samples were diluted 1 in 1000 and CSF 1 in 4 in 1% PBS: BSA. Standards, samples, and blanks were added in duplicate. Absorbance was read at 450 nM in a FLUOstar VANTI plate reader (BMG Labtech). The concentration of VCAM1 was interpolated against a standard curve, obtained by serial dilution of recombinant VCAM1 supplied with the kit (1000‐15.625 pg/mL). The mean values from duplicate measurements are provided.

### 2.9 ACE‐1 enzyme activity assay

ACE‐1 enzyme activity was measured in CSF and serum using a fluorogenic activity assay, as described previously.^15^ Serum, diluted 1 in 10, and CSF, diluted 1 in 5, was incubated with an ACE1‐specific FRET peptide substrate (10 µM) (Abz‐FRK(Dnp)‐P) (Biomol International, Exeter, UK), with and without captopril for 2.5 h at 26°C in the dark. Fluorescence was read at excitation at 320 nm and emission at 405 nm in a FLUOstar VANTI plate reader (BMG Labtech). Standards, samples, and blanks were run in duplicate, and ACE‐1 activity was determined by subtracting the fluorescence signal in the captopril‐inhibited wells from the untreated wells. A serial dilution of recombinant human ACE‐1 (1000‐31.1 ng/mL) (R&D Systems, UK) was included on each plate to maintain consistency between plates. ACE‐1 activity was expressed as relative fluorescence units (r.f.u.).

### 2.10 Statistical analysis

Each marker was measured independently across two ELISA plates, the two assays being performed on different days, and the mean values were calculated. The distribution of each dataset was visually inspected using box‐and‐whisker and Q‐Q plots generated in SPSS, and extreme outliers were removed. Independent one‐way ANOVAs with Dunnet's multiple comparisons correction were used to compare means for each of the pathology and vascular markers. Pearson correlation coefficients were calculated in SPSS to explore the relationships between CSF and serum vascular markers and markers of BBB leakiness, ADNC, and cognition. Correlation coefficients and *p* values are shown.

## 3 RESULTS

### 3.1 Demographic, pathological, and clinical features of study cohort

CSF and serum samples were collected at baseline from 75 participants in the ADNI study (Table 1; see also Table S1 for more detailed information on each participant). Cases were stratified based on clinical diagnosis and established CSF Aβ and tau cut‐off values into the following three groups: CU controls, MCI, and AD (*n* = 25/group). The age of participants at baseline did not vary between groups, nor did the gender balance. *APOE* ε4 carriers were more frequent in MCI and AD than CU controls (independent one‐way ANOVA, *p* < 0.0001). CSF t‐tau, p‐tau, and Aβ_1‐42_ (but not Aβ_1‐40_) levels differed between groups (independent one‐way ANOVA, *p* < 0.0001). The proportions of PET Aβ+ participants (*p* < 0.0001), as well as PET Aβ Centiloids and PET entorhinal tau SUVR (both *p* < 0.0001), varied significantly between groups. There were also significant between‐group differences in CDR‐M and CDR‐G scores, CDR‐SB, MoCA, MMSE, ADAS‐total, and ADAS‐13 scores (all *p* < 0.0001). The demographic, pathological, and clinical features of the study cohort are summarized in Table 1 and shown in detail in Table S1.

### 3.2 CSF markers of neurovascular injury correlate with CSF Aβ_1‐40_ and tau in early‐stage AD

**FIGURE 1:**
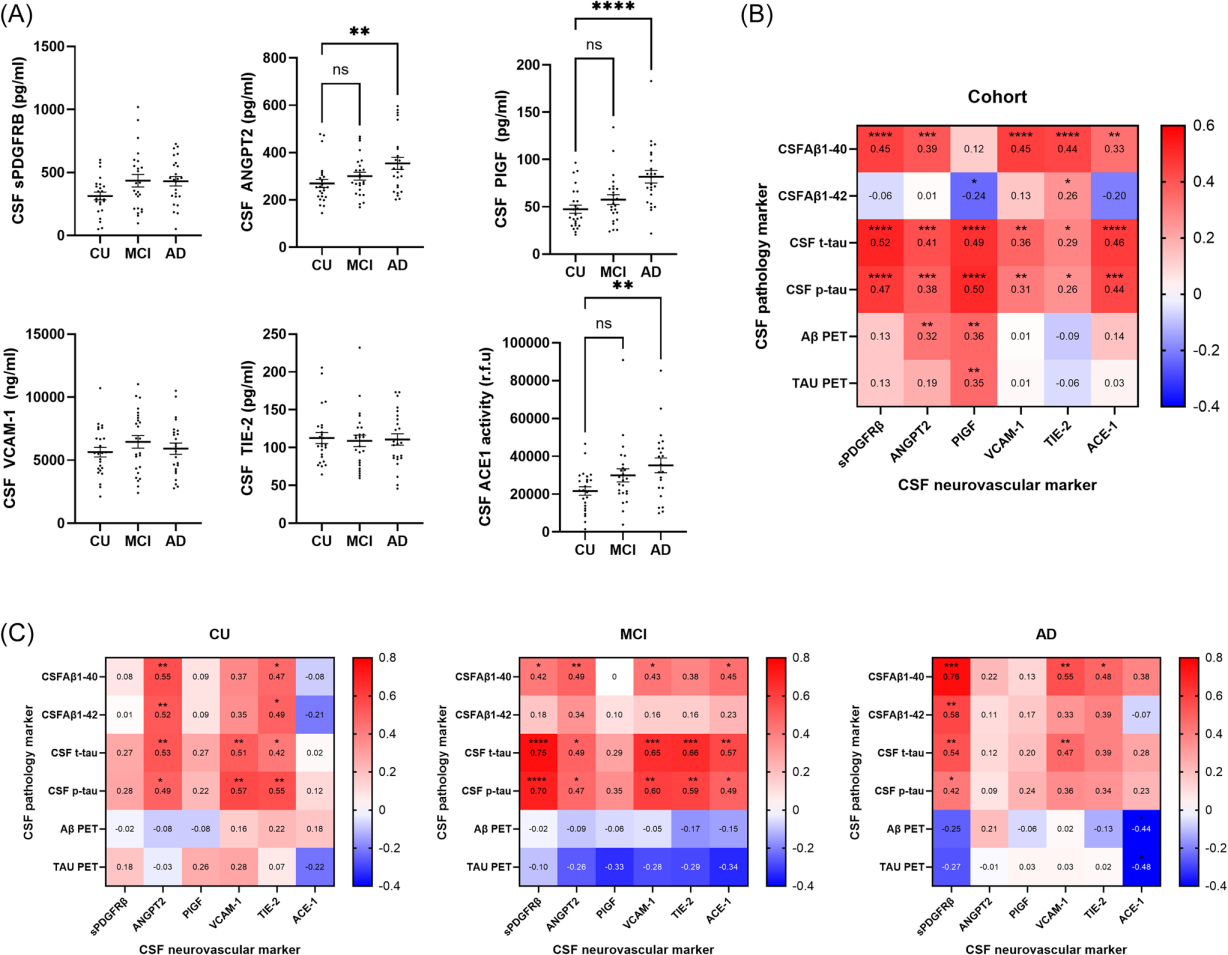
CSF levels of neurovascular markers are elevated in MCI and Alzheimer's disease (AD). (A) Scatterplots of CSF sPDGFRβ, ANGPT2, PlGF, VCAM‐1, TIE‐2, and ACE‐1 in MCI and AD compared to age‐matched cognitively unimpaired subjects (*n* = 25 per group). Independent one‐way ANOVAs were performed to compare levels of each marker across groups. Means ± SEM are shown. ns = non‐significant **p < 0.01 ****p < 0.0001. (B and C). Heatmaps showing correlation coefficients (Pearson's *r*) between CSF neurovascular markers and cognitive assessment within the combined cohort in (B) and within the independent groups in (C). *p < 0.05, **p < 0.01, ***p < 0.001, ****p < 0.0001. ACE‐1, angiotensin‐converting enzyme‐1; AD, Alzheimer's disease; ANGPT2, angiopoietin‐2; CSF, cerebrospinal fluid; MCI, mild cognitive impairment; PlGF, placental growth factor; SEM, standard error of the mean; sPDGFRβ, soluble platelet‐derived growth factor receptor beta; TIE‐2, tyrosine kinase with immunoglobulin like and EGF like domains‐2; VCAM‐1, vascular cell adhesion molecule‐1.

CSF levels of several neurovascular markers tended to be higher in MCI and AD than in CU controls. Independent one‐way ANOVAs revealed significant group differences with medium to large effect sizes (*η* ^2^ > 0.06) for PlGF (*η* ^2^ = 0.23, *p* = 0.0001), ANGPT2 (*η* ^2^ = 0.11, *p* = 0.014), and ACE‐1 (*η* ^2^ = 0.12, *p* = 0.016) and approaching significance for sPDGFRβ (*η* ^2^ = 0.08, *p* = 0.056) but not for VCAM1 or TIE‐2 (Figure 1A). Post hoc comparisons revealed that ANGPT2 (*p* < 0.01), PlGF (*p* < 0.0001), and ACE‐1 enzyme activity (*p* < 0.01) were higher in AD than controls and approached significance for sPDGFRβ in MCI versus controls (*p* = 0.06) and AD versus controls (*p* = 0.07).

We investigated the relationships between CSF neurovascular markers and CSF and PET‐imaging biomarkers of ADNC across the cohort, shown as heatmaps in Figure 1B. All vascular markers correlated positively with CSF t‐tau and p‐tau, and all, with the exception of PlGF, correlated positively with CSF Aβ_1‐40_. PlGF correlated inversely with CSF Aβ_1‐42_ and positively with PET Aβ and PET tau. CSF ANGPT2 also correlated positively with PET Aβ.

We next investigated the correlations within individual diagnosis groups, shown using heatmaps in Figure 1C. The correlation coefficient and *p* values are shown in Table S2. CSF sPDGFRβ correlated strongly with t‐tau (*r* = 0.75, *p* < 0.0001) and p‐tau (*r* = 0.70, *p* < 0.0001) in MCI and strongly with Aβ_1‐40_ (*r* = 0.76, *p* < 0.0001) and Aβ_1‐42_ (*r* = 0.58, *p* = 0.002), in addition to t‐tau (*r* = 0.54, *p* = 0.01) and p‐tau (*r* = 0.42, *p* = 0.04), in AD.

ANGPT2 correlated positively with both CSF Aβ_1‐40_ (*r* = 0.55, *p* = 0.004), Aβ_1‐42_ (*r* = 0.52, *p* = 0.008), t‐tau (*r* = 0.53, *p* = 0.007), and p‐tau (*r *= 49, *p* = 0.014) in controls. In MCI, the relationships were weaker, and ANGPT2 did not correlate with Aβ_1‐42_. No significant correlations were observed in AD. As for ANGPT2, TIE‐2 correlated positively with Aβ_1‐42_, Aβ_1‐40,_ t‐tau, and p‐tau in CU controls. In MCI, the correlations with both t‐tau (*r* = 0.66, *p* < 0.0001) and p‐tau (*r* = 0.59, *p* = 0.002) were stronger, whereas correlations with Aβ_1‐40_ and Aβ_1‐42_ were not significant. In AD, TIE‐2 correlated with Aβ_1‐40_ only.

CSF VCAM‐1 correlated positively with CSF t‐tau and p‐tau only and approached significance for both CSF Aβ_1‐40_ and Aβ_1‐42_ in CU controls. In MCI, CSF VCAM‐1 correlated strongly with t‐tau (*r* = 0.65, *p* < 0.001), p‐tau (*r* = 0.60, *p* < 0.01), and Aβ_1‐40_ (*r* = 0.43, *p* = 0.03). In AD, CSF VCAM‐1 correlated with Aβ_1‐40_ (*r* = 0.55, *p* = 0.01) and t‐tau (*r* = 0.47, *p* = 0.02).

### 3.3 CSF markers of vascular injury correlate more strongly with CSF tau in PET Aβ+ individuals

**FIGURE 2:**
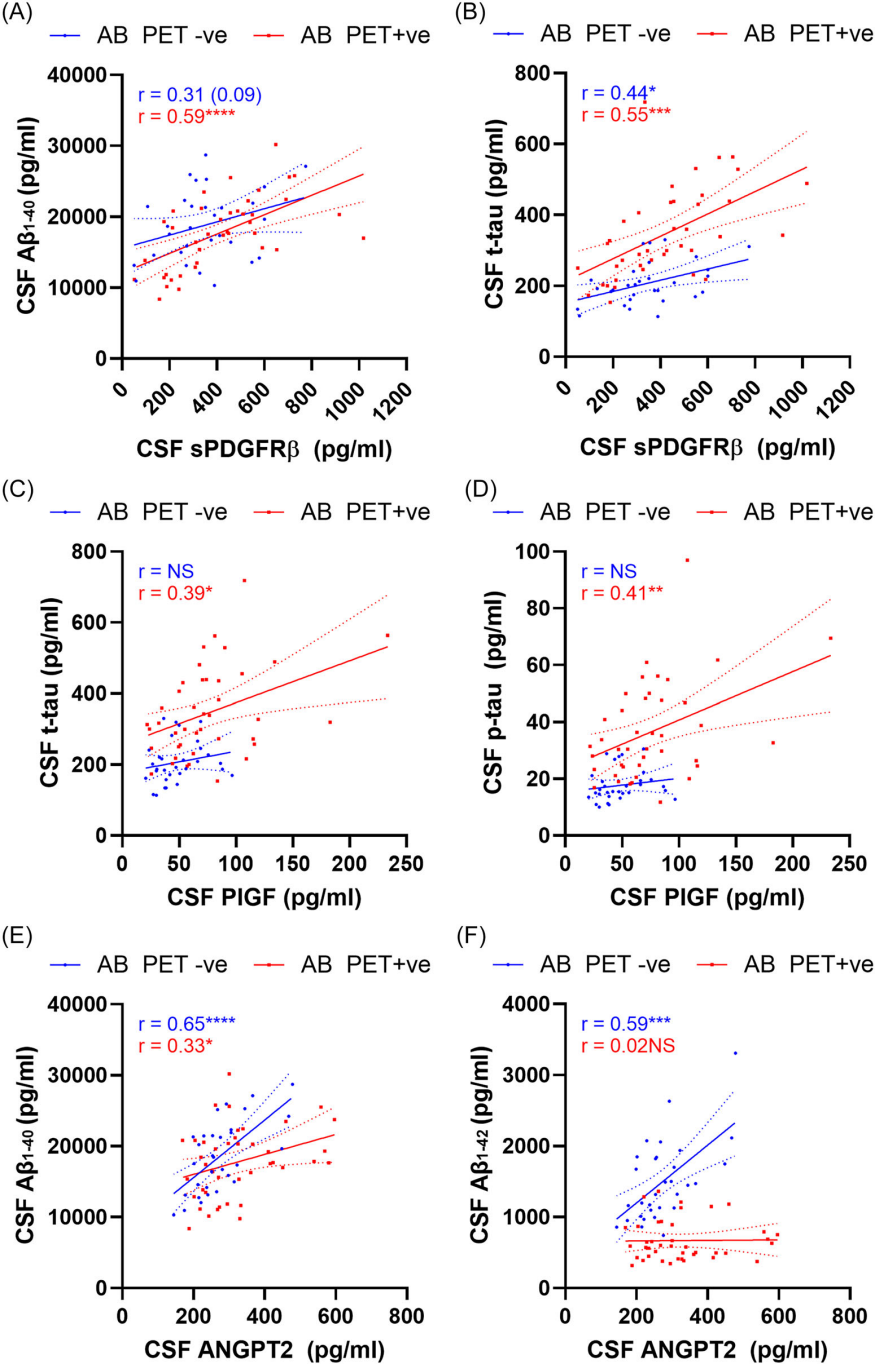
Correlation analysis showing relationships between CSF markers of neurovascular injury and ADNC in PET Aβ‐positive (Aβ+) subjects. (A–F). Scatterplots showing correlation between neurovascular markers (*x*‐axis) and CSF markers of Aβ or tau (*y*‐axis) in PET Aβ+ (red) and PET Aβ‐negative (Aβ−) (blue) participants. Linear regression lines (solid) and 95% confidence interval lines (dashed) are shown. NS = non‐significant *p < 0.05, **p < 0.01, ***p < 0.0001, ****p < 0.0001. ADNC, Alzheimer's disease neuropathological change; CSF, cerebrospinal fluid; PET, positron emission tomography.

Correlations between CSF sPDGFRβ and Aβ_1‐40_ and t‐tau across the cohort were stronger in PET Aβ+ individuals (Figure 2A‐B), as were the correlations between CSF PlGF and t‐tau/p‐tau (Figure 2C‐D). In contrast, correlations between ANGPT2 and CSF Aβ_1‐40_and Aβ_1‐42_ were stronger in the PET Aβ‐negative (Aβ−) group. The correlation coefficients and *p* values for all CSF neurovascular markers in groups stratified according to PET Aβ status are shown in Table S3.

**FIGURE 3:**
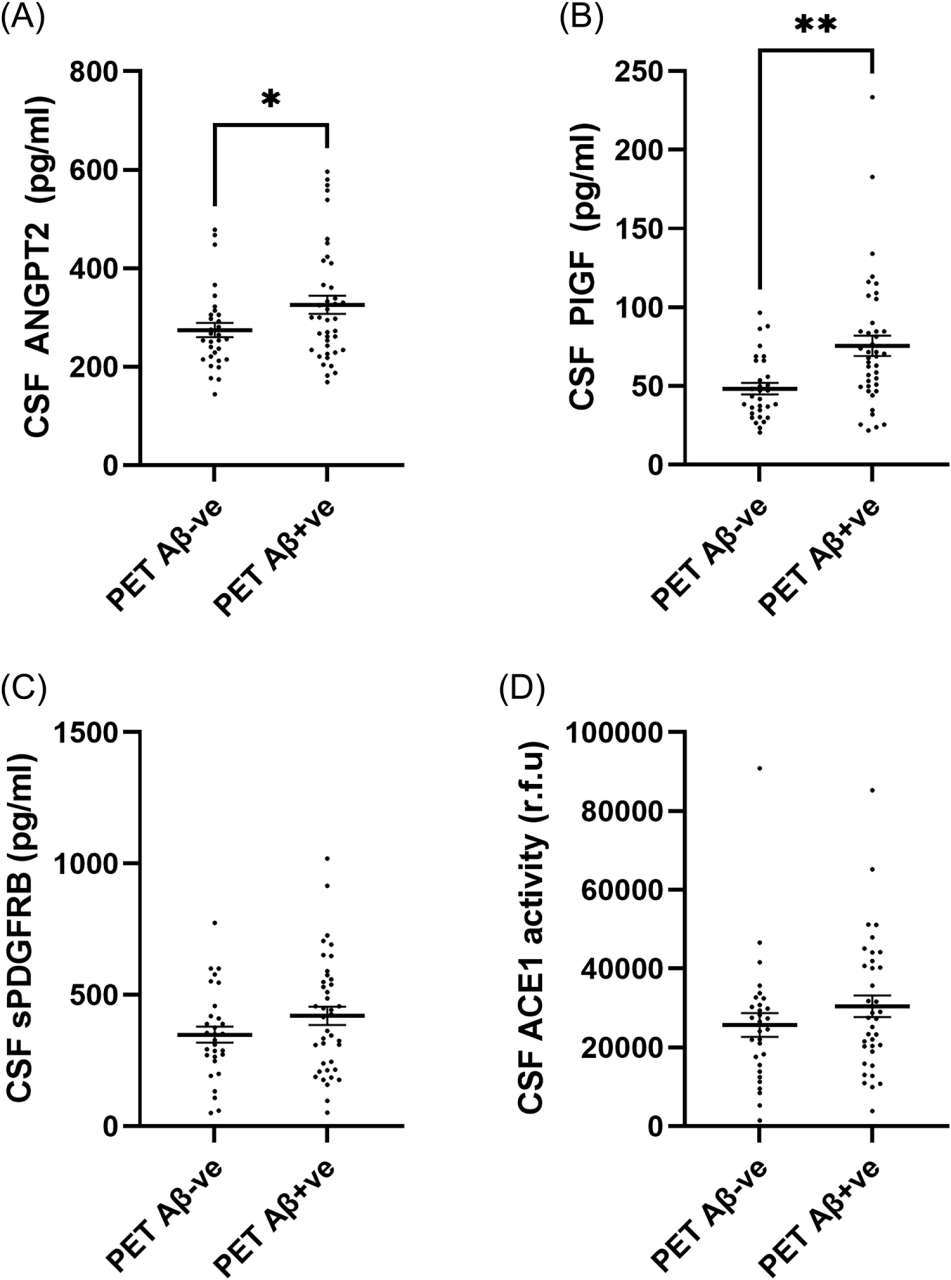
CSF levels of neurovascular injury markers are elevated in PET Aβ‐positive (Aβ+) individuals. (A–D) Scatterplots showing elevated CSF sPDGFRβ, ANGPT2, PLGF, and ACE‐1 levels in PET Aβ+ve compared to PET Aβ‐negative individuals. Means ± SEM are shown. ns = non‐significant. NS = non‐significant * *p* < 0.05, ** *p* < 0.01. ACE‐1, angiotensin‐converting enzyme‐1; ANGPT2, angiopoietin‐2; CSF, cerebrospinal fluid; PET, positron emission tomography; PLGF, placental growth factor; sPDGFRβ, soluble platelet‐derived growth factor receptor beta.

CSF ANGPT2 (*η* ^2^ = 0.058, *p* = 0.04) and PlGF (*η* ^2^ = 0.139, *p* = 0.0013) were higher in PET Aβ+ than PET Aβ− individuals (Figure 3A‐B). sPDGFRβ level and ACE‐1 activity were also numerically higher in PET Aβ+ compared to PET Aβ− individuals, but neither significantly so (Figure 3C,D). VCAM‐1 and TIE‐2 were unchanged. The mean, standard deviation (SD), and *p* value for each CSF marker in relation to PET Aβ status are shown in Table S4.

### 3.4 Serum levels of vascular markers correlate poorly with ADNC

The same panel of markers, including VEGF‐A, was measured in serum from the same donors at baseline. Serum marker levels did not vary significantly between diagnosis groups (Figure S1) and did not generally correlate with CSF or imaging markers of ADNC (Table S5). The exceptions were serum ANGPT2, which correlated positively with Aβ_1‐40_ in the combined cohort (*r* = 0.29, *p* = 0.013) and positively but weakly with Aβ_1‐40_ (*r* = 0.40, *p* = 0.046) and Aβ_1‐42_ (*r* = 0.41, *p* = 0.042) in the AD subgroup. Serum TIE‐2 correlated weakly with CSF t‐tau in AD (*r* = 0.42, *p* = 0.037) and approached significance for p‐tau and Aβ_1‐42_. Serum sPDGFRβ correlated positively with CSF Aβ_1‐42_ (*r* = 0.5, *p* = 0.015) in MCI alone.

### 3.5 CSF neurovascular injury markers PlGF and ANGPT2 correlate with cognitive decline

**FIGURE 4:**
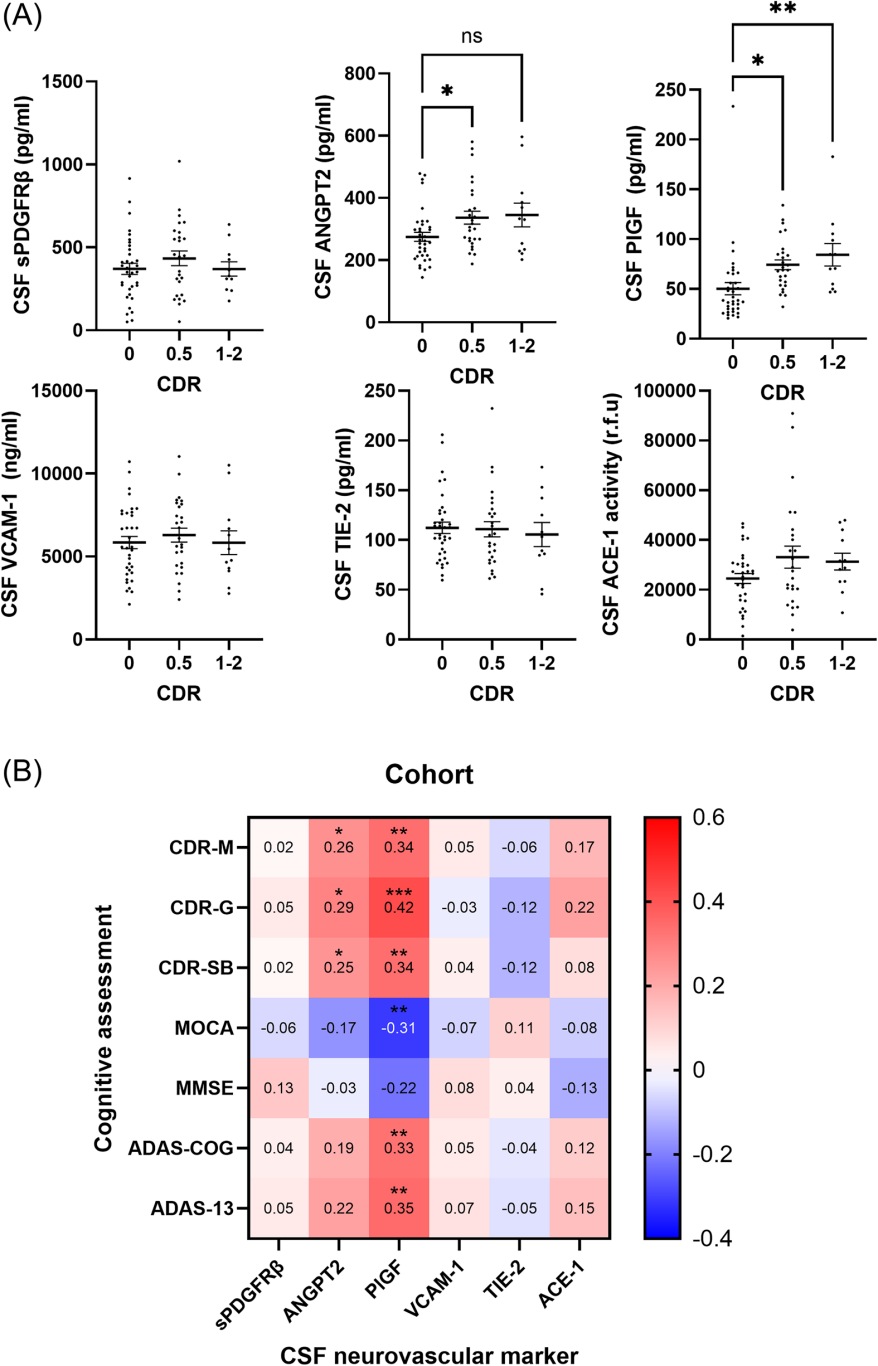
CSF levels of neurovascular injury markers are related to CDR memory. (A) Scatterplots showing levels of CSF sPDGFRβ, ANGPT2, PlGF, VCAM‐1, TIE‐2, and ACE‐1 in CDR 0 versus 0.5 versus 1‐2. Independent one‐way ANOVAs were performed to compare levels of each of marker across CDR groups. Means ± SEM are shown. ns = non‐significant. NS = non‐significant *p < 0.05, **p < 0.01 (B) Heatmaps showing correlation coefficients (Pearson's *r*) between CSF neurovascular markers and cognitive assessments within cohort. *p < 0.05, **p < 0.01, ***p < 0.0001. ACE‐1, angiotensin‐converting enzyme‐1; ANGPT2, angiopoietin‐2; CDR, Clinical Dementia Rating; CSF, cerebrospinal fluid; PLGF, placental growth factor; SEM, standard error of the mean; sPDGFRβ, soluble platelet‐derived growth factor receptor beta; TIE‐2, tyrosine kinase with immunoglobulin like and EGF like domains 2; VCAM, vascular cell adhesion molecule.

The cohort was stratified according to CDR‐M status into CDR 0, CDR 0.5, and CDR 1‐2 subgroups. Independent one‐way ANOVAs indicated that of all CSF markers, ANGPT2 (*η* ^2^ = 0.10, *p* = 0.026) and PlGF (*η* ^2^ = 0.15, *p* = 0.0026) were significantly altered in relation to CDR. Multiple‐comparisons tests revealed that CSF PlGF and ANGPT2 levels were higher in CDR 0.5 than CDR 0 (*p* < 0.05 for both) (Figure 4A). CSF PlGF was also higher in CDR 1‐2 than CDR 0 (*p* < 0.01).

We next investigated CSF markers in relation to baseline memory scores. CSF PlGF correlated positively with all three CDR domains and with ADAS‐total and ADAS‐13 scores and negatively with MoCA and approaching significance for MMSE (Figure 4B). A similar but weaker set of correlations was found for CSF ANGPT2. No significant correlations were observed for the other CSF markers. The correlation coefficient and *p* values for CSF markers are summarized in Table S6. The serum levels of the same panel of proteins, including VEGF‐A, did not correlate with the cognitive scores. The correlation coefficient and *p* values for serum markers are summarized in Table S7.

### 3.6 Markers of BBB leakiness are related to Aβ pathology and cognitive decline and are higher in Aβ+ individuals

**FIGURE 5:**
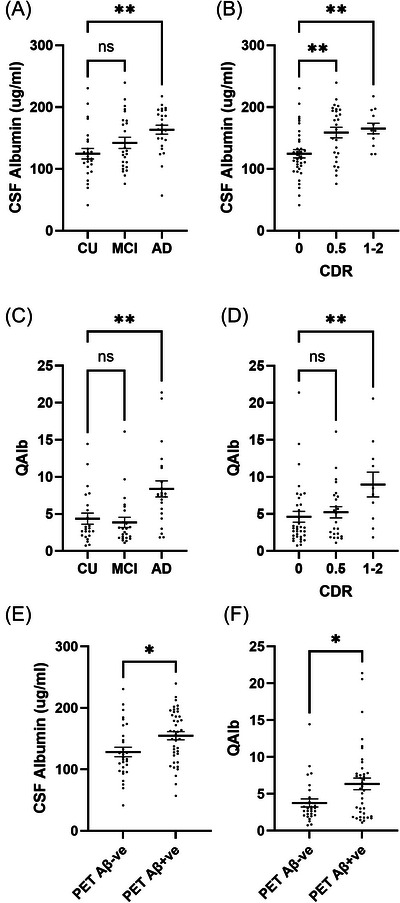
Markers of blood–brain barrier leakiness are elevated in early‐stage AD in PET Aβ‐positive (Aβ+) individuals. (A and B) Scatterplots showing CSF albumin levels in MCI, AD, and CU controls and in Clinical Dementia Rating 0, 0.5, and 1‐2. (C and D) Qalb (CSF:serum albumin ratio) in relation to diagnosis and CDR groups. (E and F) Scatterplots showing elevated levels of CSF albumin and Qalb in PET Aβ+ individuals. Independent one‐way ANOVAs were performed to assess each of the markers in relation to either diagnosis group, CDR, or PET Aβ group. Means ± SEM are shown. ns = non‐significant *p < 0.05 **p < 0.01 ****p < 0.0001. AD, Alzheimer's disease; CSF, cerebrospinal fluid; CDR, Clinical Dementia Rating; CU, cognitively unimpaired; MCI, mild cognitive impairment; PET, positron emission tomography; SEM, standard error of the mean.

CSF albumin level differed significantly between CU, MCI, and AD (*η* ^2^ = 0.13, *p* = 0.0063) and according to CDR (*η* ^2^ = 0.17, *p* = 0.0010). Multiple‐comparisons analysis showed higher CSF albumin in AD (*p* < 0.01) than CU controls and higher levels in CDR 0.5 (*p* < 0.01) and CDR 1‐2 (*p* < 0.01) than CDR 0 (Figure 5A,B). Qalb varied significantly between diagnosis groups (*η* ^2^ = 0.20, *p* = 0.0005), and according to CDR (*η* ^2^ = 0.11, *p* = 0.017), and was higher in AD than controls (*p* < 0.01) and in CDR 1‐2 than CDR 0 (*p* < 0.01) (Figure 5C‐D). CSF albumin and Qalb were higher in PET Aβ+ than PET Aβ− individuals (*p* < 0.05 for both) (Figure 5E,F).

Across the combined cohort, CSF albumin and Qalb correlated positively with PET Aβ and inversely with CSF Aβ_1‐42_. CSF albumin correlated positively with CSF t‐tau (*r* = 0.307 *p* = 0.007) and p‐tau (*r* = 0.326, *p* = 0.004), but the relationships with QAlb did not reach statistical significance. The correlation coefficients and *p* values are shown in Table S8A. CSF albumin and Qalb correlated positively with CDR scores and inversely with MoCA and MMSE across the combined cohort. Only CSF albumin correlated positively with ADAS‐total and ADAS‐13. The relationships of CSF albumin and Qalb to cognitive status are shown in Table S8B.

**FIGURE 6:**
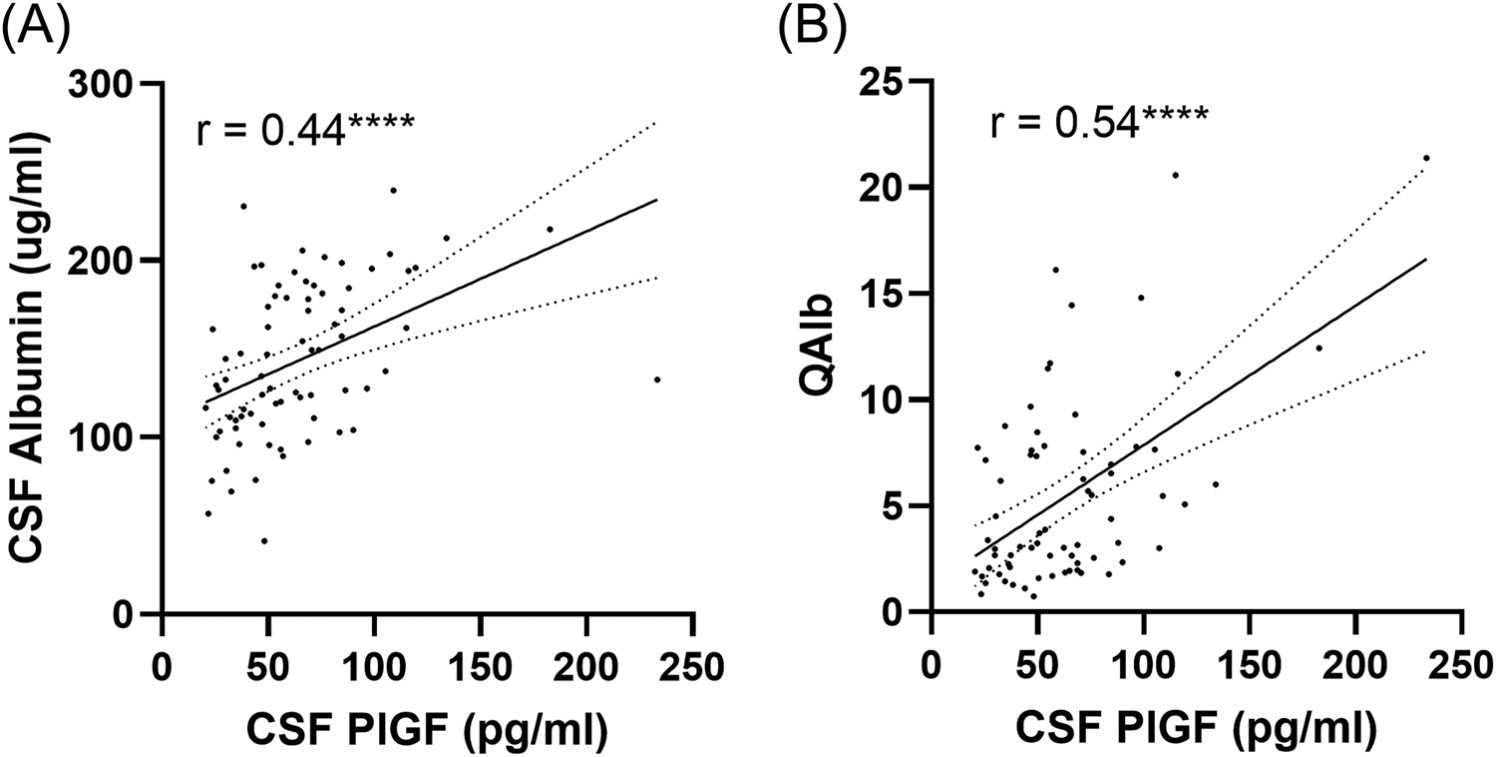
CSF PlGF is correlated with markers of BBB leakiness. Scatterplots showing correlation between CSF PlGF and (A) CSF albumin and (B) Qalb (CSF:serum albumin ratio). Linear regression lines (solid) and 95% confidence interval lines (dashed) are shown. ****p < 0.0001. BBB, blood–brain barrier; CSF, cerebrospinal fluid; PLGF, placental growth factor.

CSF albumin and QAlb correlated positively with CSF PlGF (*r* = 0.44, *p* < 0.0001 and *r* = 0.54, *p* < 0.0001) (Figure 6). CSF albumin also correlated positively with CSF ANGPT2 (*r* = 0.346, *p* = 0.002) across the entire cohort, and the correlation approached significance for PDGFRβ (*r* = 0.223, *p* = 0.059) (Table S9). CSF PlGF correlated positively with CSF albumin in MCI (*r* = 0.529, *p* = 0.007) and with Qalb in AD (*r* = 0.552, *p* = 0.006) (Table S9). Serum TIE‐2 correlated inversely with CSF albumin and had a negative association that approached significance for Qalb. However, the other serum markers did not vary with markers of BBB leakiness (Table S10).

Correlation coefficients and *p* values for the relationships between Qalb and CSF albumin, and markers of AD pathology, cognition, and CSF and serum neurovascular injury markers, in PET Aβ+ and Aβ− groups are shown in Table S11. In PET Aβ+ individuals, both Qalb and CSF albumin correlated with CSF PlGF. CSF albumin also correlated with ANGPT2. CSF albumin correlated positively with sPDGFRβ in PET Aβ− individuals.

### 3.7 Neurovascular markers are closely related to each other in CSF but not serum

**FIGURE 7:**
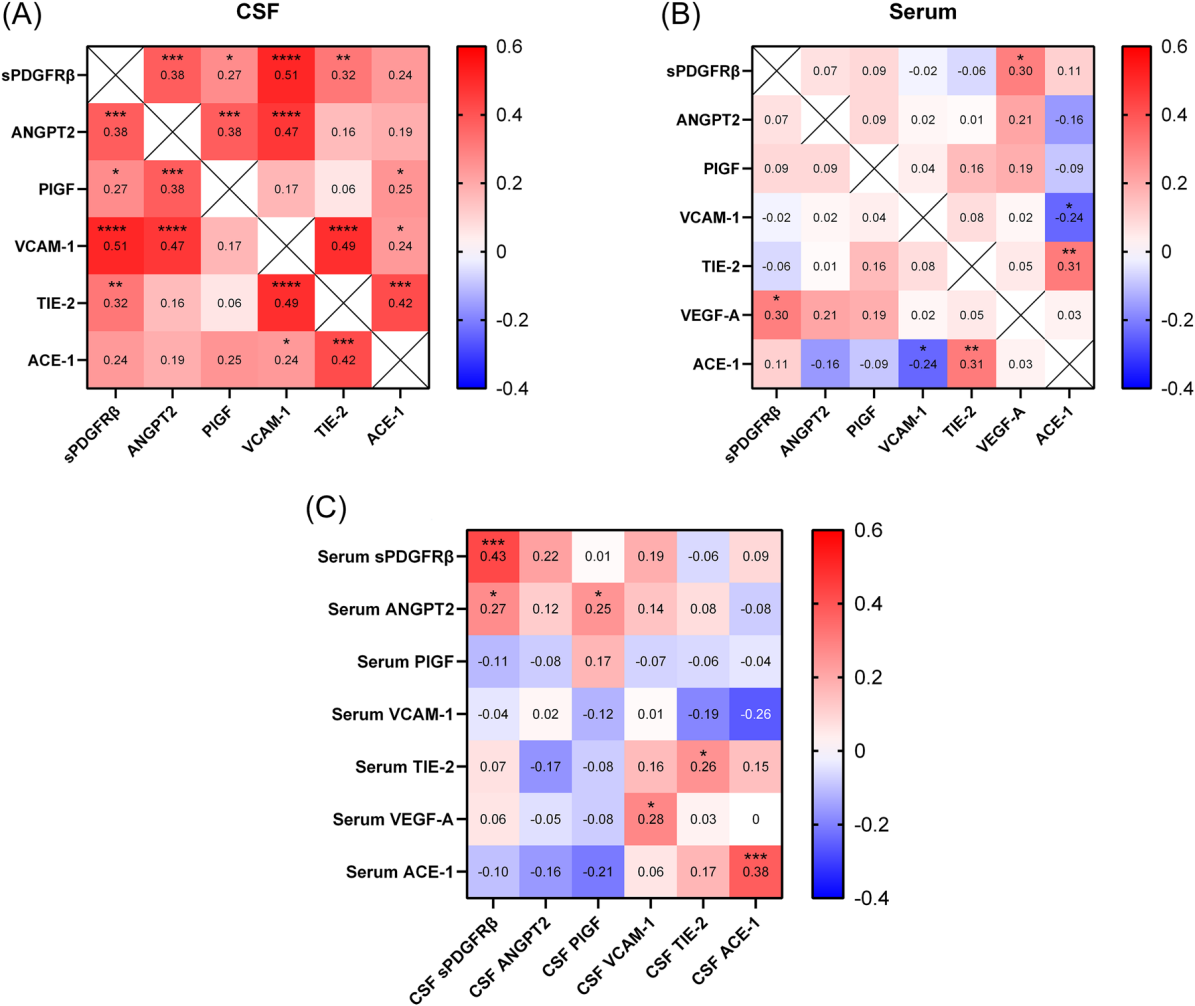
Heatmaps showing correlations between markers of neurovascular injury in CSF and serum. Correlations between neurovascular markers in (A) CSF and (B) serum markers. (C) Correlations between matched neurovascular markers in serum and CSF. CSF, cerebrospinal fluid.

There were multiple positive correlations in CSF between the different markers of vascular damage: sPDGFRβ, ANGPT2, PlGF, VCAM‐1, TIE‐2, and ACE‐1 activity (Figure 7A). Significant interrelationships were fewer and weaker for serum (Figure 7B). Levels of sPDGFRβ, TIE‐2, and ACE‐1 activity in CSF correlated positively with those in matched serum samples (Figure 7C). Correlation coefficients, *n* numbers, and *p* values are shown in Table S12.

## 4 DISCUSSION

Findings in this study confirm previous studies showing that elevated CSF markers of neurovascular injury are closely associated with markers of BBB leakiness, tau pathology, and cognitive decline. These relationships, starting in the presymptomatic stages of AD, were stronger in PET Aβ+ individuals. CSF levels of PlGF, ANGPT2, and sPDGFRβ were elevated from early‐stage AD, that is, CDR 0.5, and were strongly related to CSF Aβ_1‐40,_ t‐tau, p‐tau, and cognitive decline across multiple domains: CDR, MoCA, MMSE, and ADAS. PlGF correlated particularly strongly with Qalb, a marker of BBB leakiness. The data support previous evidence of a close relationship between markers of neurovascular injury, tau pathology, and cognitive decline that is associated with BBB leakiness starting in the presymptomatic stages of AD. Serum levels of the same panel of markers did not generally mirror findings in CSF and were largely unrelated to disease pathology or cognition, with the exception of weak correlations of serum ANGPT2 and TIE‐2 with pathology or cognitive decline that warrant further investigation in larger independent cohorts.

All CSF markers of neurovascular injury included in this study correlated with CSF t‐tau and p‐tau. This builds on previous findings, including our own, showing that CSF levels of the pericyte marker sPDGFRβ^16^, ^17^, ^18^, ^19^, ^20^, ^22^ and CSF levels of endothelial injury, including VEGF‐A, ICAM‐1, VCAM‐1,^13^ ANGPT2,^20^ and cadherin,^29^ correlate with CSF t‐tau and p‐tau in early AD. In this study, CSF sPDGFRβ and PlGF correlated more strongly with CSF tau in PET Aβ+ individuals. Stronger relationships between CSF endothelial markers, VEGF‐A, ICAM‐1, and VCAM‐1, and CSF‐tau,^14^ and CSF sPDGFRβ and tau,^30^ were previously reported in PET Aβ+ individuals. A recent study showed that CSF p‐tau levels correlated strongly with a panel of angiogenesis markers,^23^ indicating that vascular remodeling and tau are closely related. Tau oligomers accumulate within endothelial cells in *post mortem* AD brain tissue^31^ and drive microtubule destabilization associated with the induction of endothelial senescence in a mouse model of tauopathy (P301S).^32^ Fibrillar tau shifts endothelial cell metabolism toward glycolysis, inducing inflammation and BBB leakiness in young P301S mice.^33^ Together, these data suggest that vascular injury and tau pathology are closely related and start in early‐stage AD, particularly in people who are Aβ+, that is, on the AD pathway. The relevance of vascular injury mapping to changes in tau is that tau oligomers and tangles correlate more closely with the onset of cognitive decline in AD compared to Aβ.^34^ Mediation analysis has revealed that neuroinflammation mediates the association between sPDGFRβ and markers of synaptic remodeling and neurodegeneration.^20^ We previously showed that CSF ANGPT2 was also strongly related to CSF markers of neuroinflammation (YKL‐40 and TREM‐2) and neurodegeneration (t‐tau and α‐synuclein),^21^ suggesting that similar pathways are involved.

Our in‐group analysis of the relationships in CU, MCI, and AD groups indicated that the relationships between CSF markers of vascular injury and AD pathology differ according to the stage of disease. CSF sPDGFRβ correlated most strongly with t‐tau and p‐tau in MCI, a finding in keeping with other recent studies.^17^, ^18^, ^20^ In AD alone, CSF sPDGFRβ also correlated strongly with CSF Aβ_1‐40_ and Aβ_1‐42_. Lv et al. also reported that CSF sPDGFRβ correlated with CSF t‐tau and p‐tau in CDR 0.5 individuals and additionally correlated with both CSF tau and Aβ_1‐42_ in CDR 1‐2.^17^ Elevated CSF sPDGFRβ in AD is thought to reflect ADAM‐10‐mediated shedding of cell‐surface PDGFRβ from pericytes^13^ in response to Aβ and tau, as demonstrated in vitro and in vivo (reviewed in Kim et al.^2^ and Alcendor^35^). The relative contributions of Aβ, tau, and other unidentified precipitants of pericyte degeneration may differ in MCI and established AD.

The ANGPT/TIE signaling pathway regulates vascular and BBB stability – elevated gene *ANGPT2* expression^10^ and higher CSF ANGPT2 levels^21^ were previously reported in AD. In this study, CSF ANGPT2 and soluble TIE‐2 correlated strongly with CSF tau in CU and MCI but not in AD. We previously showed that CSF ANGPT2 is elevated in MCI.^21^ In the present study, we similarly found that ANGPT2 level was elevated in CDR 0.5 compared to CDR 0. In contrast to sPDGFRβ and PlGF, CSF ANGPT2 correlated more strongly with t‐tau and p‐tau in PET Aβ− individuals. These findings may indicate that altered ANGPT2/TIE signaling occurs independently of Aβ accumulation or is more strongly related to tau pathology. A previous study reported that elevation of CSF sPDGFRβ levels in CDR 0.5 was independent of Aβ and tau pathology,^13^ indicating that Aβ and tau‐independent pathways are responsible for cleavage or secretion of vascular receptors and markers under pathological conditions.

Among the panel of neurovascular markers that we studied, PlGF was the most strikingly elevated in AD, and the increase was evident from CDR 0.5, that is, at an early disease stage. As for the other markers, CSF PlGF correlated with CSF t‐tau and p‐tau, but uniquely, CSF PlGF level also correlated inversely with CSF Aβ_1‐42_ and positively with PET Aβ and PET‐tau. PlGF levels were elevated in PET Aβ+ individuals, and the strength of correlation between CSF PlGF and CSF tau was influenced by Aβ status. CSF PlGF also correlated strongly with Qalb, a measure of BBB leakiness, and with cognitive assessments across all domains. These findings might indicate that additional factors, such as BBB leakiness, mediate the relationship between PlGF and cognitive decline in AD. This would be in keeping with the reported elevation of CSF PlGF in vascular dementia (VaD), Parkinson's disease, and frontotemporal dementia (FTD),^25^ as well as in AD. Elevated CSF PlGF was associated with increased white matter hyperintensity (WMH) volume, independent of Aβ status, in CU and MCI.^36^ These findings indicate that vascular contributions to BBB leakiness and cognitive decline may occur independently of Aβ and tau pathology in AD.

Qalb, a gold‐standard measure of BBB integrity,^37^ was elevated in AD and was significantly higher in PET Aβ+ individuals. Qalb correlated with lower CSF Aβ_1‐42_ and higher PET Aβ levels, whereas the association with PET‐tau did not reach significance. The findings are consistent with other evidence that Aβ and tau contribute to BBB leakiness. Qalb is elevated in other diseases that cause dementia, including Dementia with Lewy bodies, VaD, and FTD, and in type 2 diabetes.^38^ CSF markers of endothelial injury, such as VEGF‐A, ICAM‐1, and VCAM‐1 were associated with elevated Qalb and are also raised in other dementia‐associated diseases, indicating that these changes may not be AD specific. CSF sPDGFRβ was also shown to correlate with Qalb in MCI‐AD,^18^, ^20^ although we did not replicate this in the present study, in which CSF PlGF was the only marker to correlate with Qalb. CSF markers cannot provide insights into brain regional variations in BBB integrity and may not be sensitive enough to detect BBB leakiness limited to specific brain regions, such as BBB breakdown in the hippocampus detected in CDR 0.5 individuals by high‐contrast MRI.^12^, ^13^

Peripheral markers of neurovascular injury would offer a non‐invasive, less expensive alternative to CSF lumbar puncture. A recent longitudinal study revealed that accelerated cognitive decline in people with cerebral Aβ was associated with lower plasma VEGF‐A and higher PlGF, in turn associated with accelerated accumulation of neocortical tau.^39^ Plasma and serum PlGF levels are also associated with cerebrovascular injury and WMH severity in AD.^40^, ^41^ In the present study, serum levels of most neurovascular markers tested, including VEGF‐A and PlGF, did not correlate strongly with their CSF counterparts or with markers of ADNC and cognitive decline. This is likely to reflect multiple factors, including differences in brain: CSF, CSF:serum, and BBB permeability and/or transport; extracranial contributions to serum neurovascular markers; and their stability, cellular interactions, and peripheral metabolism.

Weak but significant correlations were observed between serum ANGPT‐2 and Tie‐2 and ADNC: serum ANGPT2 correlated positively with CSF Aβ_1‐40_, and serum TIE‐2 correlated positively with CSF t‐tau in AD. Serum TIE‐2 also correlated inversely with markers of BBB leakiness – Qalb and CSF albumin. Serum and CSF PDGFRβ levels were positively correlated, supporting our previous observation in matched CSF and serum from healthy volunteers.^16^ Serum and CSF Tie‐2 and ACE‐1 activity were also positively correlated. These weak correlations may reflect the direct release of some neurovascular markers into both the CSF and also directly into the bloodstream across a damaged BBB. Although the present data largely reflect a limited association between neurovascular markers in CSF and serum, larger independent studies are required to rule out some of the weaker but significant findings that indicate a potential link between peripheral markers of ANGPT/TIE and sPDGFRβ signaling in relation to ADNC, BBB leakiness, and cognitive decline.

The major limitations of the study are its cross‐sectional design and relatively small cohort size, which limited us to exploring the relationships of baseline markers of neurovascular injury (in CSF and serum) to markers of ADNC and cognition. Follow‐up studies to explore causality in larger longitudinal studies will be needed to clarify the temporal relationships between disease pathology, the different CSF neurovascular markers, and changes in cognition and to confirm some of the weaker relationships between serum markers, neurovascular function, and disease. Despite its limitations, this study provides further evidence that CSF markers of vascular dysfunction and BBB leakiness track closely the development of tau pathology and cognitive decline, particularly in Aβ+ individuals, from very early stages of disease. The study also highlights PlGF as a mediator and potential biomarker of cerebrovascular injury and BBB breakdown associated with cognitive decline from early in the development of AD. Larger independent studies are required to confirm the validity of some of the weaker relationships observed for serum markers of neurovascular injury.

## CONFLICT OF INTEREST STATEMENT

The authors declare no conflicts of interest. Author disclosures are available in the Supporting Information.

## CONSENT STATEMENT

All participants included in this study provided informed consent.

## Supporting information



Supporting Information

Supporting Information

## Data Availability

Data have been uploaded to the ADNI database and can be accessed via an ADNI data use agreement
