# Supplementary material for: CSF markers of vascular injury correlate with tau and cognitive decline in early Alzheimer's disease
Source: Alzheimers Dement. 2025 Nov 30;21(12):e70957. doi: 10.1002/alz.70957 (PMC12665166; doi:10.1002/alz.70957)
Supplement: Supplementary file 1 — Supporting Information [file ALZ-21-e70957-s002.docx]

**Supplementary material:**

**Table S1**

**Detailed demographic, pathological, and cognitive data for all individuals within the study.** CU = cognitively unimpaired; MCI = mild-cognitive impairment; AD = Alzheimer’s disease. Age = Age at baseline assessment. Sex - 1 = Male, 2 = Female. Diagnosis – 1 = control, 2 = MCI, 3 = AD. Aβ40 = CSF Aβ_1-40_ (pg/ml); Aβ42 = CSF Aβ_1-42_ (pg/ml); t-tau = CSF total tau (pg/ml); p-tau = CSF tau-p181 (pg/ml). APOE genotype. PET Aβ = centiloids. PET Tau = SUVR entorhinal cortex. CDR-M – clinical dementia rating memory ; CDR-G – clinical dementia rating global; CDR-SB – clinical dementia rating sum-of-boxes. MoCA – Montreal Cognitive assessment. MMSE – Mini-mental state examination. ADAS-COG – Alzheimer’s disease assessment scale. ADAS-13 is the expanded version of ADAS-COG with inclusion of 2 additional tasks.

**Table S2.**

**Correlation analysis between CSF markers of neurovascular injury and CSF and imaging markers of Alzheimer’s disease neuropathological change (ADNC).** Pearson’s correlation coefficients (r), p-values, and number of participants included for each set of analysis (N) are shown. Alzheimer’s disease (AD). Significant correlations are shown in bold. Correlations approaching significance are underlined. P < 0.05 was used to identify significant correlations.

**Table S3.**

**Correlation analysis between CSF markers of neurovascular injury (top row) and CSF and imaging markers of Alzheimer’s disease neuropathological change (ADNC) in PET Aβ-ve and PET Aβ+ve individuals.** Pearson’s correlation coefficients (r), p-values, and number of participants included for each set of analysis (N) are shown. Alzheimer’s disease (AD). Significant correlations are shown in bold. Correlations approaching significance are underlined. P < 0.05 was used to identify significant correlations.

**Table S4.**

CSF neurovascular markers in relation to PET Aβ status (0 = PET Aβ-ve; 1 = PET Aβ+ve). Means and standard deviation (SD) are shown. Markers that are significantly elevated in PET Aβ+ve group are highlighted in bold.

**Figure S1.**


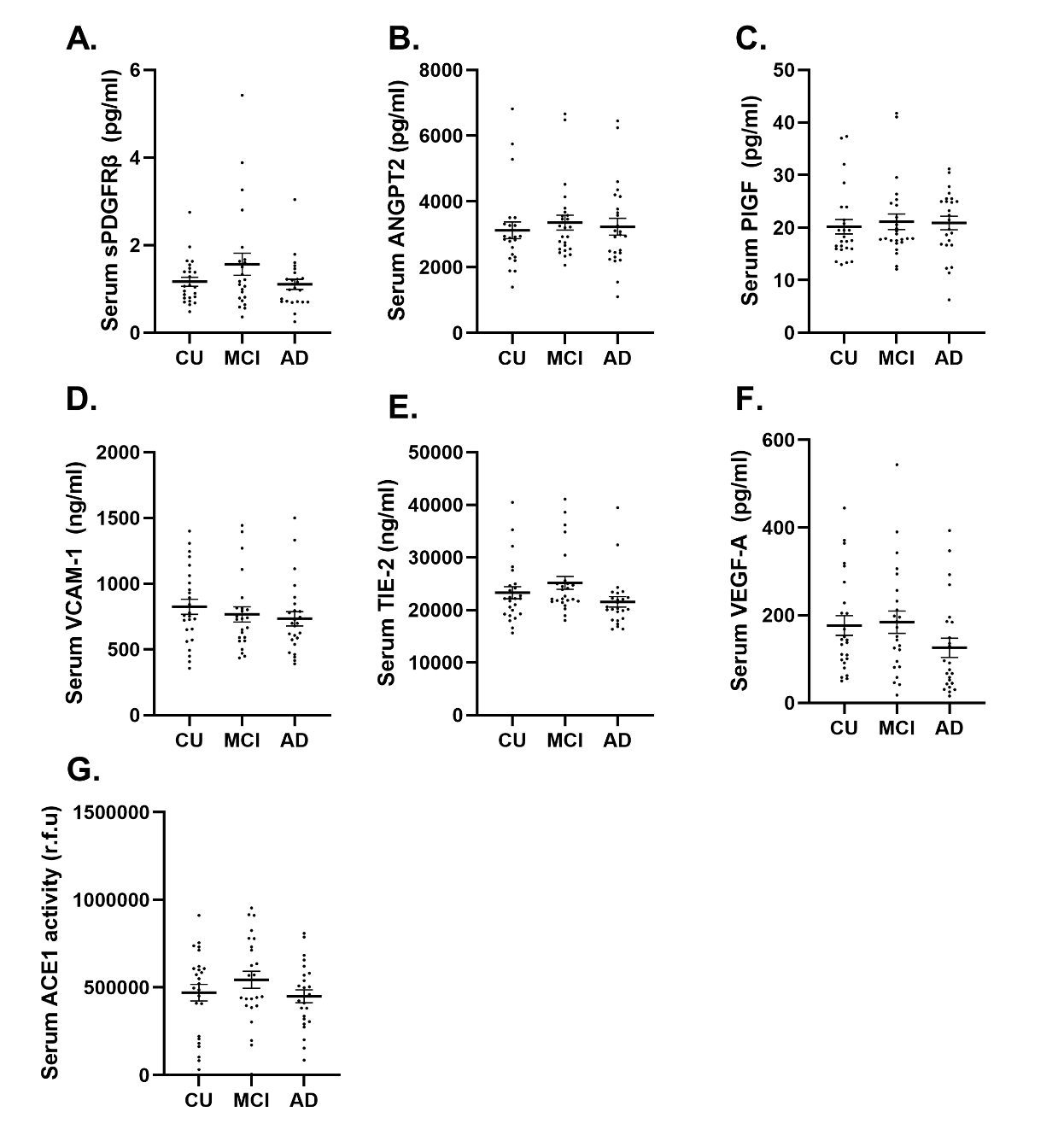


**Figure 1. Serum levels of neurovascular markers were unalerted in cognitively unimpaired (CU), mild-cognitively impaired (MCI), and Alzheimer’s disease (AD) (n = 25 per group).** **A-G** Scatterplots of soluble platelet-derived growth factor receptor β (sPDGFRβ), angiopoitein-2 (ANGPT2), placental-like growth factor (PLGF), vascular cell adhesion molecule (VCAM), Tyrosine Kinase with Immunoglobulin and Epidermal Growth Factor (EGF) Homology Domains 2 (TIE-2), vascular endothelial growth factor-A (VEGF-A) and angiotensin-converting enzyme-1 (ACE1) across groups. Means ± SEM are shown.

**Table S5.** **Correlation analysis between serum markers of neurovascular injury and CSF and imaging markers of Alzheimer’s disease neuropathological change (ADNC).** Pearson’s correlation coefficients (r), p-values, and number of participants included for each set of analysis (N) are shown. Linear correlation analysis was performed across the entire cohort and within the individual diagnosis groups: cognitively unimpaired (CU), mild-cognitively impaired (MCI) and Alzheimer’s disease (AD). Significant correlations are shown in bold. Correlations approaching significance are underlined. P < 0.05 was used to identify significant correlations.

**Table S6.**

**Correlation analysis between CSF markers of neurovascular injury and cognitive assessment across the cohort**. CDR = clinical dementia rating for memory (M), global (G) and sum-of-boxes (SB); MOCA = Montreal Cognitive assessment; MMSE = mini-mental state examination; ADAS = Alzheimer’s disease assessment scale.

**Table S7.**

**Correlation analysis between serum markers of neurovascular injury in relation to markers of cognition.** Pearson’s correlation coefficients (r), p-values, and number of participants (N) are shown for each analysis. Linear correlation was performed across the entire cohort and within the individual diagnosis groups: Cognitively unimpaired (CU) controls; mild-cognitive impairment (MCI) and Alzheimer’s disease (AD). Significant correlations are shown in bold. Correlations approaching significance are underlined. P < 0.05 was used to identify significant correlations.

**Table S8.**

**Correlation analysis between markers of BBB leakiness, ADNC and cognition.** (A) Analysis of Qalb ratio and CSF albumin levels in relation to CSF and imaging markers of ADNC. (B) Analysis of Qalb and CSF albumin in relation to assessment of cognitive decline: clinical dementia rating (memory, global, and sum of boxes), Montreal Cognitive assessment (MOCA), Mini-mental state examination (MMSE), and Alzheimer’s disease assessment scale (ADAS-Cog and ADAS-13). Pearson’s correlation coefficients (r), p-values, and number of participants (N) are shown for each analysis. Linear correlation was performed across the entire cohort. Significant correlations are shown in bold. Correlations approaching significance are underlined. P < 0.05 was used to identify significant correlations.

**Table S9.**

**Correlation analysis between CSF markers of neurovascular injury and markers of BBB leakiness.** Pearson’s correlation coefficients (r), p-values, and number of participants (N) are shown for each analysis. Linear correlation was performed across the entire cohort and within the sub-groups (cognitively unimpaired – CU, mild-cognitive impairment – MCI, and Alzheimer’s disease – AD). Significant correlations are shown in bold. Correlations approaching significance are underlined. P < 0.05 was used to identify significant correlations.

**Table 10.**

**Correlation analysis between serum markers of neurovascular injury and markers of BBB leakiness.** Pearson’s correlation coefficients (r), p-values, and number of participants (N) are shown for each analysis. Linear correlation was performed across the entire cohort and within the sub-groups (cognitively unimpaired – CU, mild-cognitive impairment – MCI, and Alzheimer’s disease – AD). Significant correlations are shown in bold. Correlations approaching significance are underlined. P < 0.05 was used to identify significant correlations.

**Table 11.**

**Correlation analysis between markers of BBB, and ADNC, cognition and CSF and serum markers of neurovascular injury, in relation to PET Aβ status.** Pearson’s correlation coefficients (r), p-values, and number of participants (N) are shown for each analysis. Linear correlation was performed across the entire cohort: (A) shows correlation analysis between BBB markers and CSF and imaging markers of ADNC in groups stratified by PET Aβ status. Significant correlations are shown in bold. Correlations approaching significance are underlined. P < 0.05 was used to identify significant correlations.

**Table 12.**

**Correlation analysis between CSF and serum markers of BBB, and ADNC, cognition and CSF and serum markers of neurovascular injury.** Pearson’s correlation coefficients (r), p-values, and number of participants (N) are shown for each analysis. Linear correlation was performed across the entire cohort: (A) shows correlation analysis between CSF markers, (B) shows correlations analysis between serum markers and (C) shows correlation analysis between CSF and serum markers. Significant correlations are shown in bold. Correlations approaching significance are underlined. P < 0.05 was used to identify significant correlations.
